# The Influence of the Hours of the Day on Mortality

**Published:** 1851-09

**Authors:** 


*The influence of the hours of the day on mortality.*—The observations and calculations of Dr. Casper lead to the conclusion that the maximum of deaths occurs in the fore part of the day, and the mininum between evening and midnight. The explanation of this, Dr. Casper seeks in the analogy between sleep and death: sleep, being the period in which great organic changes occur, is *pro tanto*, favorable to the dissolution of the individual.

The diseases which are the causes of death exert a modifying influence on the hour at which death occurs. The origin and progress of diseases, their exacerbations, and remissions, are frequently observed to occur at certain times of the day. The influence of these on the hours of death is shown by the following table of 5591 deaths from various diseases.

| ACUTE DISEASES. | From midnight to 6 A. M. | From 6 A .M. to noon. | From noon. to 6 P. M. | From 6 P M. to midnight. |
|---|---|---|---|---|
| Fevers | 64 | 75 | 66 | 66 |
| Inflammations | 160 | 164 | 182 | 160 |
| Exanthemata | 44 | 45 | 43 | 59 |
| CHRONIC DISEASES. | | | | |
| Phthisis | 186 | 240 | 215 | 186 |
| Atrophy | 347 | 381 | 282 | 255 |
| Hæmorrhages | 163 | 186 | 161 | 121 |
| Chronic Catarrh | 41 | 47 | 34 | 24 |
| Dropsies | 90 | 119 | 93 | 64 |
| Neuroses | 267 | 267 | 191 | 179 |
| Other chronic Diseases | 76 | 102 | 89 | 85 |
| Totals { Acute | 268 | 284 | 291 | 285 |
| Totals { Chronic | 1140 | 1344 | 1065 | 914 |

The next table exhibits the variations from the general rule, in the same class of diseases, on the side either of excess or deficiency, as indicated by the signs + or —.

| | From midnight to 6 A. M. | From 6 A. M. to noon. | From noon to 6 P M. | From 6 P. M. to midnight. |
|---|---|---|---|---|
| ACUTE DISEASES | | | | |
| Fevers . . . . . . . | —16 | —15 | + 1 | +30 |
| Inflammations . . . . | —12 | —45 | +31 | +26 |
| Exanthemata . . . | —22 | —55 | —18 | +95 |
| CHRONIC DISEASES | | | | |
| Consumption . . . | —27 | — 1 | +17 | +11 |
| Atrophy . . . . . . | + 5 | +18 | —15 | — 8 |
| Hæmorrhages . . . . | + 6 | + 4 | +12 | —22 |
| Chronic Catarrh . . . | +29 | +31 | —10 | —50 |
| Dropsies . . . . . . | — 6 | +17 | +11 | —33 |
| Neuroses . . . . . . | +43 | + 6 | —32 | —17 |
| Other Chronic Diseases . | —36 | — 1 | +10 | +27 |

The following are briefly the conclusions of Dr. Casper on the influences investigated by him :—

1. *As to births.*—More births occur from nine o'clock in the evening to six o'clock in the morning than during other hours of the twenty-four. Labor-pains commence more frequently between midnight and three o'clock in the morning than at other times. Of those births which terminated during the day, the majority were male children. Labor is longer if the pains begin in the day-time than if it commence during the night. This influence is more striking with still-born than with living children.

2. *As to deaths.*—The maximum general mortality occurs during the earlier hours of the day, the mininum in the evening. Of special causes of death the relative mortality with reference to the time of day presents many variations. Inflammatory diseases present their maximum in the after-part of the day; fevers and exanthemata in the earlier hours of the night; hæmorrhages in the fore part of the day and in the afternoon; and the neuroses generally in the hours after midnight.—*London Med. Gaz. April* 1851.

